# The cGAS-STING/PERK-eIF2α: Individual or Potentially Collaborative Signaling Transduction in Cardiovascular Diseases

**DOI:** 10.7150/ijbs.101247

**Published:** 2024-10-28

**Authors:** Xueqi Wan, Huan Zhang, Jinfan Tian, Libo Liu, Ziyu An, Xin Zhao, Lijun Zhang, Xueyao Yang, Changjiang Ge, Xiantao Song

**Affiliations:** 1 Department of Cardiology, Beijing Anzhen Hospital, Capital Medical University, Beijing Institute of Heart, Lung and Blood Vessel Disease, Beijing 100029, P.R. China.; 2 Department of Radiology, Beijing Anzhen Hospital, Capital Medical University, Beijing Institute of Heart, Lung and Blood Vessel Disease, Beijing 100029, P.R. China.

**Keywords:** cGAS-STING, PERK-eIF2α, UPR, CVDs, therapeutics

## Abstract

Over the past several decades, a canonical pathway called the cyclic GMP-AMP (cGAMP) synthase (cGAS)-stimulator of interferon genes (STING) mediating type I interferon (IFN) release via TANK-binding kinase 1(TBK1) / IFN regulatory factor 3 (IRF3) pathway has been widely investigated and characterized. Unexpectedly, recent studies show that the cGAS-STING noncanonically activates the protein kinase RNA-like ER kinase (PERK)-eukaryotic initiation factor 2α (eIF2α), an essential branch of unfolded protein response (UPR), even before the activation of the TBK1/IRF3 signaling. Additionally, we found that the PERK could regulate the STING signaling besides being modulated by upstream cGAS-STING. However, earlier evidence solely focused on the unidirectional regulation of STING and PERK, lacking their functional crosstalk. Hence, we postulate that there is a complex relationship between the cGAS-STING and PERK-eIF2α pathways and that, through convergent downstream signaling, they may collaboratively contribute to the pathophysiology of cardiovascular diseases (CVDs) via the cGAS-STING/PERK-eIF2α signaling axis. This study provides a novel pathway for the development of CVDs and paves the foundation for potential therapeutic targets for CVDs.

## Introduction

Cardiovascular diseases (CVDs), including atherosclerosis (AS), cardiomyopathy, and myocardial infarction (MI), are prevalent global health concerns. These diseases are associated with high morbidity and mortality 1-3. The mechanisms underlying the pathogenesis and progression of CVDs are complicated and include biological processes such as cellular proliferation, migration, apoptosis, necrosis, ferroptosis, pyroptosis, macrophage phenotype switching, inflammation, and more 4-6. Broadly, a constellation of signal transduction pathways determine cellular phenotypes, and aberrant signaling activation associated with CVDs leads to pathological changes and poor clinical prognosis 7.

Prior research has demonstrated that the cyclic GMP-AMP (cGAMP) synthase (cGAS)-stimulator of interferon genes (STING) signaling pathway, which is a conventional immunological and inflammatory system, is associated with numerous CVDs 8-10. In the traditional signaling pathway, cGAS senses DNA misplaced in the cytosol and responds by synthesizing cyclic guanosine monophosphate adenosine monophosphate (cGAMP), which binds to STING, a small molecule located in the endoplasmic reticulum (ER). This interaction results in the conformation and movement of STING to the ER-Golgi membrane and the subsequent recruitment of TANK-binding kinase 1 (TBK1). TBK1 then phosphorylates IRF3. IRF3, which acts as a transcription factor, elevates the expression of type I interferon (IFN-Is)-stimulated genes (ISGs). The activation of TBK1 also recruits IκB kinase-ε (IKKε), followed by the nuclear factor kappa beta (NF-ΚB) activation. Next, type I interferons (IFN-Is) and other proinflammatory cytokines are promptly released and initiate a series of reactions to defend against the invasion of microbial or other pathogens 11, 12. The cGAS-STING-TBK1-IRF3 axis has been thoroughly studied and verified as a primary and standard pathway that is involved in a series of diseases. As the connections between STING and ER stress are close, and the PERK is indispensable for the ER stress signaling transduction, there has been a growing focus on the regulation of cGAS-STING for the PERK-eIF2α pathway 13-15.

Protein kinase RNA-like ER kinase (PERK), which is localized to the ER, is a kinase capable of detecting ER distress and then halting translation to prevent protein misfolding. This process is known as the unfolded protein response (UPR). Under normal circumstances, PERK's lumen domain is tethered to glucose-regulated protein (GRP78) and silenced. However, in cases of ER disorder, PERK can detach from GRP78 and undergo phosphorylation, ensuring the initiation of downstream signaling pathways 16-19. More recent research has also shown that PERK is closely associated with the cGAS-STING pathway 20. The cGAMP-stimulated STING can also activate PERK, preceding TBK1-IRF3 initiation. This STING-activated PERK then interacts with eukaryotic initiation factor 2α (eIF2α) and participates in a variety of biological activities in the ER 20. PERK-mediated phosphorylation of eIF2α induced by ER stress and other stimulation ultimately culminates in the attenuation of protein biogenesis and potential cell death 21. It is reported that the PERK-eIF2α pathway plays a crucial role in CVD pathology 42, 113, 131. The phosphorylation of eIF2α enables the translation of specific mRNAs that encode activating transcription factor 4 (ATF4), which is an essential transcriptional factor for regulating redox reactions and amino acid metabolism. Excessive levels of ATF4, in turn, dephosphorylate eIF2α by upregulating the protein phosphatase 1 regulatory subunit (e.g., the C terminus) of the growth arrest and DNA damage gene *GADD34* to restore protein synthesis and maintain ER protein homeostasis 17, 22, 23. Remarkably, the PERK-eIF2α complex also partially regulates cGAS-STING signaling, which is essentially involved in host-pathogen interactions. Previous evidence has shown that STING can also be activated via non-canonical pathways that are independent of cGAS conjunction 24, 25. Additionally, PERK plays a crucial role in regulating STING alone, leading to its translocation and the activation of downstream cascades 26-28.

While several studies have highlighted potential regulatory relationships between the cGAS-STING and PERK-eIF2α pathways, they primarily illustrate a one-way modulation rather than a reciprocal interaction. Both pathways appear to significantly influence various CVDs. However, existing research has largely focused on the actions of individual pathways, neglecting a comprehensive understanding of the combined roles of the cGAS-STING and PERK-eIF2α pathways in CVDs.

We propose that a complex interplay exists between cGAS-STING and PERK-eIF2α signaling, and their potential mutual interactions may contribute to various pathological changes associated with CVDs. Investigating the connections between these pathways and their effects on different CVDs could reveal novel mechanisms and therapeutic targets, ultimately informing more effective clinical treatments.

## 1. The individual or potentially collaborative interplay of the cGAS-STING/PERK-eIF2α signaling

### 1.1 Overview of the cGAS-STING pathway

The cGAS-STING pathway is intimately involved in an array of immunological and inflammatory responses, which partially contribute to the pathogenesis and development of many diseases 29, 30. The initial step of this pathway is the cGAS sensing misplaced DNA from exogenous (e.g., extracellular) microbe or endogenous (e.g., from the mitochondria) cell 31, 32. The cGAS then binds to double-stranded DNA (dsDNA), allosterically increasing its catalytic activity and giving rise to the production of cGAMP, a second messenger molecule that activates STING 30, 33. STING, which is a type of DNA adaptor that is located in the ER, consists of a transmembrane (TM) domain with four TM helices, a cytoplasmic ligand-binding domain, which is necessary for dimerization and cGAMP binding, and a C-terminal tail which hosts the TBK1 phosphorylation site that is needed to initiate its downstream cascade 34. Under normal conditions, STING forms a domain-swapped homodimer and is oligomerized upon cGAMP stimulation. This activated STING then translocates from the ER through the ER-Golgi intermediate compartment (ERGIC) to the Golgi with the assistance of coatomer protein complex II (COPII) vesicles 35, 36. Upon reaching the ERGIC and Golgi compartments, STING can recruit TBK1 and facilitate its autophosphorylation, which is followed by the phosphorylation and nuclear relocation of IRF3 and NF-κB 11, 31, 37. IRF3 enables the activation of a series of target genes, including interferon-stimulated genes (ISGs), and NF-κB contributes to the expression of inflammation-related factors, including interleukin (IL)-6 and IL-12 29. The newly released IFNs, ILs, and other cytokines instigate immune and inflammatory responses. Although the activation of cGAS-STING is crucial in the defense of pathogens or viruses, the excessive activation of STING could lead to autoimmune diseases. Therefore, the activated cGAS-STING cascade needs to be restrained to maintain the immune homeostasis of the cell. Emerging evidence has elucidated that several elements of autophagy are involved in the suppression of STING signaling, such as autophagy-related (ATG) 9A and LC3 38, 39. CSNK1A1/CK1α, a serine/threonine protein kinase, is responsible for impeding the overactivation of STING-mediated type I IFN signaling via promoting autophagic degradation of STING 40. In addition to being eliminated by the autophagic process, cGAS-STING also participates in the orchestration of autophagy, which contributes to the obliteration of foreign nucleic acids in the host cell cytosol. Earlier research demonstrated that autophagy could be induced by STING trafficking, independent of TBK1 and downstream signaling. An in-depth investigation has found that upon cGAMP stimulation, STING translocates to the ERGIC, which serves as a source for the LC3 lipidation, resulting in the formation of autophagosomes. Notably, sea anemone, a sort of extremely ancient species, was uncovered to be able to induce LC3 conversion in reaction to cGAMP, while its STING structure lacked the C-terminal TBK1 activation domain, indicating that the autophagy activation could be the primordial and intrinsic function of the cGAS-STING pathway 41. Overall, this signaling axis allows the cGAS-STING complex to regulate a broad repertoire of cellular defense mechanisms.

### 1.2 Overview of the PERK-eIF2α pathway

PERK, which is localized in the ER, is the pivotal branch point that mediates the UPR. It is highly vulnerable and capable of being activated in response to persistent ER stress caused by disturbances in aberrant calcium levels, hypoxia, glucose deprivation, or the accumulation of unfolded and misfolded proteins. The UPR is a series of cellular responses that are triggered by certain stimuli- including ER stress or increases in misfolded proteins—and which stops translation and aids correct protein folding by up-regulating molecular chaperones and/or triggering cellular apoptosis 42. There are three key branches orchestrated by membrane-related sensors in the UPR process: inositol-requiring enzyme 1(IRE1α), PERK, and ATF6. First, PERK can oligomerize and phosphorylate eIF2α, which reduces cellular protein synthesis and prompts the translation of the ATF4. Second, IRE1 RNase can splice X-box binding protein (XBP1) mRNA and produce the activated transcription factor XBP1. Thirdly, the ATF6 translocates from the ER to the Golgi apparatus, where it is cleaved by site-1 protease and site-2 protease to release an NH2-terminal domain that enters into the nucleus to promote the related gene expression 22, 43, 44. Under normal states, PERK binds with chaperone proteins such as immunoglobulin heavy chain-binding protein (BiP) (also known as glucose-regulated protein 78 (GRP78) to form complexes to be silenced 45. When faced with certain stressors, PERK dissociates from its complex and is activated. The specific downstream signaling pathway that PERK activates depends on the intensity of the stressors. Under low-intensity ER stress, the PERK branch mainly relieves the ER protein folding burden by phosphorylating eIF2α, impeding the translation of certain proteins, and mobilizing transcription factor 4 (ATF4) and other proteins. Under high-intensity ER stress, hyperactivated PERK can facilitate the higher expression of C/EBP-homologous protein (CHOP), which reduces the expression of the gene encoding antiapoptotic BCL-2 and dictates cellular demise 45-48.

eIF2 serves as the major hub for inducing translational repression. It is composed of three main subunits: α, β, and γ. This tripolymer constitutes a ternary complex with guanosine triphosphate (GTP) and initiator-methionyl-tRNA (Met)-tRNAi 49. The tetramer can combine with the 40s subunit of ribosomes and be incorporated (along with other eukaryotic initiation factors) to create the 43S preinitiation complex. Afterward, the 43S complex recognizes the AUG initiation codon and forms the larger 48S complex. The process requires GTP as an energy source. Hydrolysis of GTP frees eIF2-GDP and other eIFs from the ribosome, allowing them to conduct their subsequent functions. The phosphorylation of eIF2α at ser 51 is a major checkpoint for the suspension of protein synthesis 45, 49, 50. Generally, eIF2α phosphorylation is accomplished by the assembly of four kinases: PERK, protein kinase double-stranded RNA-dependent (PKR), general control non-derepressible-2 (GCN2), and heme-regulated inhibitor (HRI) 51. In adaptative responses, the phosphorylation of eIF2α minimizes cellular toxicity by reducing protein burdens and boosting the transcription of selective genes, while its prolonged activation elicits cell death via CHOP 52-54. It is reasonable to hypothesize that eIF2α phosphorylation can either lead to cell death or survival depending on the duration and degree of the subsequent signaling cascade. However, non-phosphorylated eIF2α is ubiquitinated and degraded by the E3 ubiquitin ligase carboxyl terminus of the HSC70-interaction protein (CHIP) in the absence of chaperone proteins, which upregulates cyclic AMP-dependent transcription factors. The eIF2α phosphorylation can also block ubiquitination 55. Once ER homeostasis is restored, p-eIF2α levels return to their normal baseline (which is dominated by PP1) 54, 56.

### 1.3 Overview of the cGAS-STING/PERK-eIF2α pathway

#### 1.3.1 Crosstalk among STING, ER-stress, and UPR

As discussed earlier, the ER stress and UPR are closely associated with the PERK activation. Besides, as an essential DNA adapter located in the ER, STING is tightly linked to the ER stress response, thus suggesting an underlying crosstalk among STING, ER stress, and UPR 57. It is widely accepted that mtDNA plays an indispensable role in the activation of the cGAS-STING pathway and connects with ER stress 10, 58. Chronic ER stress and ISG expressions have been observed under autoimmune conditions. Furthermore, this gene expression was offset by the deficiency of STING or UPR branches, including PERK and ATF6, where the mtDNA acts as a mediator. Overall, the ER stress set in motion UPR chains, leading to the mtDNA release and ROS production, followed by the cGAS-STING signaling cascade and IFN release 59. Existing evidence has unveiled that the function of STING is not completely dependent on the IFN-related pathways. To explore the mechanisms of how STING primes T-cell death, Wu *et al.* established the STING^N153S/+^ mouse model and revealed that these mutants could trigger chronic activation of ER stress and UPR, leading to T-cell apoptosis. Moreover, the ER stress inhibitor impedes T-cell death. Further experiments verified that the motif-helix aa322-343, called the “UPR motif”, which is different from the domain that is necessary for setting off IFN, is required for inducing UPR. These findings suggest that certain stimulators can interact with the UPR motif of STING and instigate ER stress and UPR 57. Additionally, due to the innate immune function of STING, its deficiency can lead to infection. It has been shown that Seneca Valley Virus infection leads to immune escape via the degradation of reticulophagy mediated by PERK and ATF6, however, the intrinsic mechanisms remain unclear 60. Besides immunological effects, the STING-NOD-like receptor protein 3 (NLRP3) mediates inflammatory responses 29, 61. Interestingly, during Brucella abortus infection, the STING promotes inflammasome formation via UPR point IRE1-α. The IRE1-α activation facilitates mitochondrial ROS (mROS) release, stabilizes hypoxia-inducible factor-1 (HIF-1), and enhances inflammation in macrophages 62. These findings were consistent with the previous conclusion that ROS production could be an upstream trigger for the NLPR3 activation 63. Additionally, a study by Lu *et al.* has shown that the inhibition of NLRP3 inflammasome decreases ROS production, suggesting a feedback loop between ROS and the NLRP3 inflammasome formation 64. ROS is closely associated with ER stress. Previous studies have shown that ER stress activates NLRP3 inflammasome formation 65. It is reported that contact sites between the ER and mitochondria-associated ER membranes (MAM) are crucial for various cellular activities. The calcium imbalance causes ER stress and NLRP3-mediated inflammation via MAM 66. Therefore, it is reasonable to speculate that STING, ER stress, and UPR may coordinately orchestrate the inflammation activation. These consequences may indirectly suggest that there may be complicated crosstalk between STING, ER, and UPR during inflammation and immunity-related processes, where the ROS and MAM might be crucial mediators.

#### 1.3.2 The cGAS-STING/PERK-eIF2α interaction

As mentioned above, the cGAS-STING pathway mediates canonical immunological and inflammatory responses via TBK1-IRF3 activation and IFN release, which is intimately associated with various diseases, including CVDs. Notably, recent studies have delineated that the cGAS-STING also mediates the activation of the PERK-eIF2α, even prior to the activation of the TBK1-IRF3. PERK-eIF2α is one of the cascade branches orchestrating ER stress-induced UPR. Its activation generally hinders the production of proteins and specifically enhances the transcription of particular mRNAs, which can contribute to the development of various illnesses. Additionally, previous evidence has shown a robust link between the downstream effects of the cGAS-STING pathway and ER stress-related signaling 14, 15, 57, which also indirectly indicates a certain relationship between cGAS-STING/PERK-eIF2α.

Existing evidence has elucidated the probable connections between STING and PERK. Notably, Zhang *et al.* 20 visualized the interaction between PERK and STING using proximity ligation assays and domain-mapping assays after co-immunoprecipitation and determined a direct domain interaction between STING and PERK. Mechanistically, the STING C-terminal domain fusion continuously elevated the PERK kinase domain's autoactivation capacity. Surprisingly, a previous study demonstrated that, during viral infections, STING promoted IFN release and inhibited protein synthesis in an IFN-independent manner. However, the intrinsic mechanism remains unknown. PERK was then confirmed to intertwine with STING and be activated by STING 67. Another article demonstrated that PERK activation constrained viral replication by promoting STING-induced IFN release 68. These experiments indicate that the STING-orchestrated and PERK-mediated pathways interact with each other during immune-mediated inhibition of viral replication. Additional data supports the notion that STING can be modulated by PERK, although not all studies come to this conclusion. As one example, eliminating PERK thwarted Nrf2 and promoted mitochondrial disfunction and mtDNA accumulation. Subsequently, STING and its downstream effectors were stimulated to enhance IFN release and increase anti-tumor effects 26. Another paper showed that PERK phosphorylation inhibited the translocation of STING, resulting in the immunosuppression of myeloid-derived suppressor cells (MDSCs) 69. By contrast, in another paper, inhibiting PERK quenched the STING-mediated classical route and IFN production in neurons after traumatic brain injury (TBI), thus alleviating brain injury and cell loss 27. Likewise, in macrophages infected by M. bovis, PERK activation promoted both the translation of STING and its downstream signaling. Phosphorylated IRF3 is shown to initiate apoptosis in three ways: entering the nucleus to trigger the transcription of apoptotic genes, associating with Bax to induce the mitochondrial apoptotic pathway, and activating caspase 8 70. Therefore, the regulation between STING and PERK is mostly positive, and the controversies about the interactions between PERK and STING may have partially arisen from differences in the cell types or pathological contexts studied.

**Figure 1:**
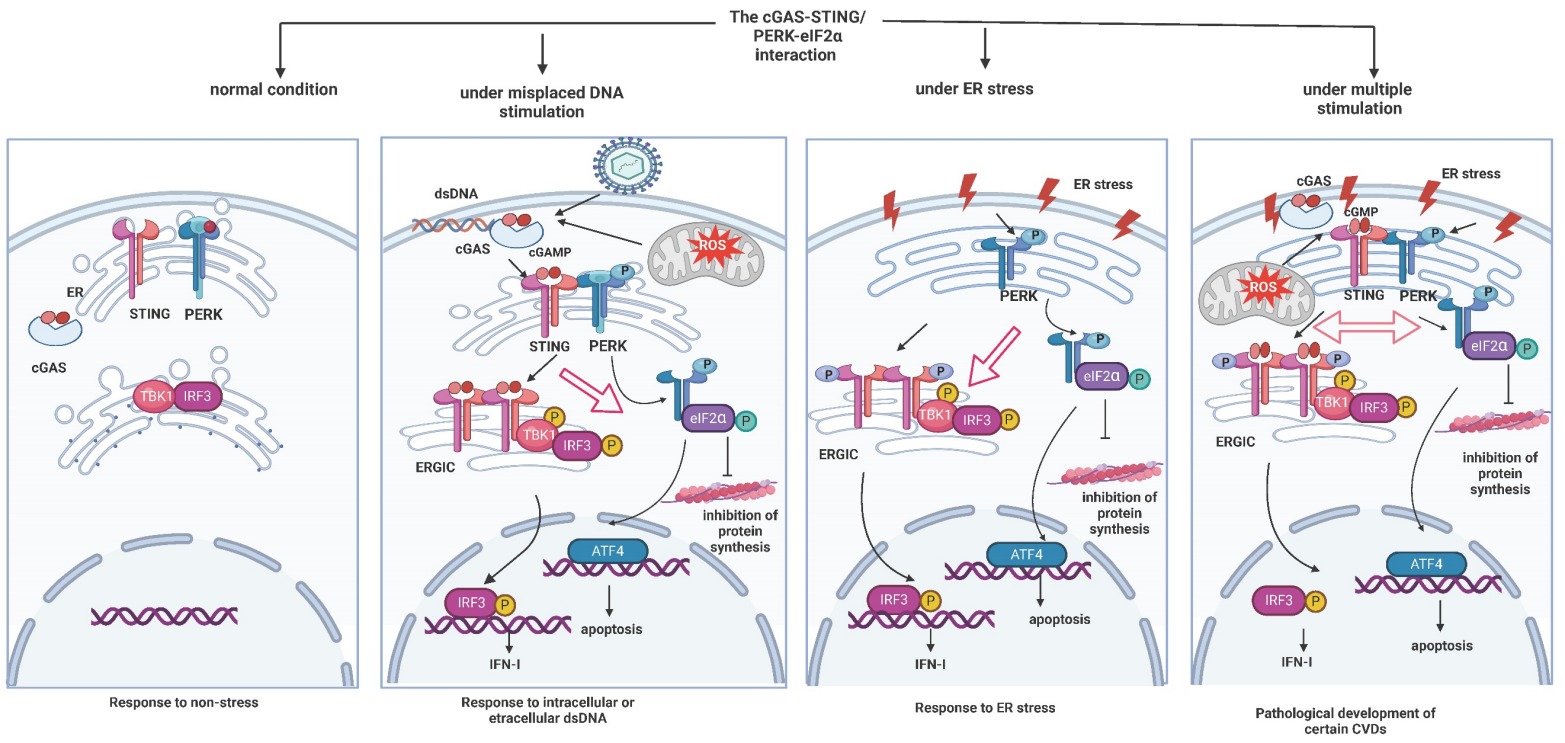
cGAS-STING/PERK-eIF2α interactions under different conditions (created with BioRender.com). Under normal conditions, both the cGAS-STING pathway and ER stress-related PERK-eIF2α pathways are in states of silence. Upon stimulation from leaked DNA, either from exogenous pathogens or intracellular mitochondria, STING that is localized to the ER can bind with cGAMP produced by cGAS to be activated and translocated to the ER-Golgi intermediate compartment (ERGIC). This translocation promotes downstream TBK1 and IRF3 phosphorylation to mediate IFN release. STING remains at the ER to interact with PERK and activate it, which inhibits overall protein synthesis. In turn, in times of ER stress, PERK is phosphorylated and facilitates the activation of eIF2α and ATF4, simultaneously interacting with STING. This interaction promotes STING's translocation to ERGIC to induce the TBK1 and IRF3 cascade. Under complex pathological conditions, both the cGAS-STING axis and the PERK-eIF2α axis are activated. Interactions between STING and PERK are also augmented, meaning that the activation of either molecule can promote the initiation of the other to mediate the pathological effects.

The sum of the evidence suggests that the cGAS-STING pathway and the PERK-eIF2α pathway may undergo reciprocal regulation and that a combined cGAS-STING/PERK-eIF2α pathway may arise to function in pathological contexts. Rather than unidirectional regulation, we surmise that STING and PERK collaborate to affect other cellular pathways and undergo mutual regulation. We also propose a probable mechanism underlying the cGAS-STING/PERK-eIF2α interaction (**Figure 1**): Under normal conditions, the cGAS-STING pathway and ER-stress-related PERK-eIF2α pathways are both inactive. However, upon stimulation from DNA leakage, ER-localized STING can activate PERK and thus inhibit overall protein synthesis. In turn, ER-stress-induced phosphorylated PERK simultaneously interacts with STING to promote its translocation to ERGIC, where it triggers the TBK1 and IRF3 cascades. Therefore, both activated STING and activated PERK can regulate each other. Studies have shown that inhibiting PERK does not affect the STING-mediated canonical pathways, which might be result from STING signaling initiated by other potent stimulators. In summary, cross-regulation between STING and PERK is intricate, and cGAS-STING/PERK-eIF2α positive crosstalk plays a vital role in various pathological conditions.

## 2. The cGAS-STING pathway in CVDs (Table 1)

### 2.1 The role of the cGAS-STING in atherosclerosis (AS)

An array of mechanisms, including inflammatory responses, endothelial disruption, lipid accretion, macrophage phenotype conversion and foam cell formation, aberrant smooth muscle cells (SMC) proliferation, and migration 4, 5, 71, 72, are involved in the pathogenesis and development of AS. Mounting evidence has shown that the cGAS-STING pathway is involved in each of these cellular activities and thus accelerates AS. DNA leakage is the primary stimulus for the cGAS-STING activation. Increases in cytosolic cell-free DNA and mtDNA have been observed in AS patients compared with the general population. Further analysis has shown that cigarette smoke leads to the deposition of plasma DNA, the initiation of the cGAS-STING pathway, and the release of inflammatory factors like IL-6, which may be a crucial mechanism underlying AS progression 9. IQ motif-containing GTPase-activating protein 1 (IQGAP1) is thought to be an essential scaffolding protein that modulates mitochondrial function and affects endothelial cell behavior. IQGAP1 expression levels increase in high-fat diet (HFD)-triggered AS models in apolipoprotein E-deficient ApoE^-/-^ and Ldlr^-/-^ mice models. Mechanistically, the mitochondrial dysfunction indirectly activates the cGAS-STING and NLRP3-directed pyroptosis. Furthermore, IQGAP1 knockdown reverses the activation of this signaling pathway. To explore the underlying mechanism, human umbilical vein endothelial cells (HUVECs) were treated with palmitic acid (PA) to construct an *in vitro* AS model. Results show that silencing either IQGAP1 or cGAS-STING attenuated endothelial injury in AS by decreasing the lactate dehydrogenase (LDH) activity, cytokine factor secretion, and cellular pyroptosis 5. Transactive response DNA-binding protein of 43 kDa (TDP43) is a protein that combines with DNA and is involved in nucleic acid metabolism, including the release of mitochondrial DNA. It also acts as an activator of the cGAS-STING 73, 74. TDP43 induces cGAS-STING by promoting the mtDNA release and NF-κB signaling, exacerbating lipid absorption in macrophages, and eventually encouraging foam cell accumulation. As expected, the genetic knockout of TDP43 in macrophages decreased the expression of pro-inflammatory markers and lipid uptake. Eliminating TDP43 in AS mice also blunted lesion areas 75. Even in the context of chronic kidney disease (CKD), the cGAS-STING pathway plays a significant role in the development of atherosclerotic (AS) plaques. In CKD models, heightened oxidative stress induces mitochondrial damage, which activates cGAS-STING signaling. This activation led to phenotypic switching, premature senescence, and increased plaque instability. Notably, the depletion of either cGAS or STING resulted in reduced plaque areas and diminished plaque instability 10. These results indicate that the cGAS-STING pathway drives the progression of AS by affecting cell inflammation, pyroptosis, lipid deposition, and SMC phenotypic switching.

### 2.2 The Role of cGAS-STING in Cardiomyopathy

The cGAS-STING signaling is involved in various kinds of cardiomyopathy. Many factors can contribute to the development of cardiomyopathy, including lipid toxicity, obesity, pathological states like diabetes mellitus (DM), infectious diseases, chemical drugs, and hereditary mutations. Each of these factors can cause disruptions in cardiac contractile or diastolic functioning 58, 76-79. Obesity is a common risk factor for a constellation of CVDs, including but not limited to myocardial dysfunction. Recent work showed that a STING-mediated NLRP3 pathway was hyperactivated in the HFD-induced obese mice. Eight weeks of aerobic exercise could ameliorate STING-NLRP3-induced inflammation and pyroptosis and improve cardiac function, but these improvements were abolished after the injection of a STING agonist. These results demonstrate that suppressing STING can protect against obesity-related cardiopathy to some extent 76.

Diabetic cardiomyopathy (DCM) is a fairly common complication of DM 78. The cardiac function is undermined by DM because the pathological changes augmented cardiac fibrosis and pyroptosis, which are partially driven by the cGAS-STING pathway. A specific polypeptide, Irisin, played a protective role in the DCM by blocking the cGAS-STING signaling. Additionally, initiation of the STING pathway partially offsets Irisin's favorable effects. Inactivation of cGAS-STING signaling, however, prevented cardiomyocyte disruption by raising sensitivity to autophagy in both high-glucose-stimulated H9C2 cells and DCM mice. Mechanistically, the liver kinase B1 (LKB1)/adenosine monophosphate-activated protein kinase (AMPK)/Unc-51-like autophagy activating kinase 1 (ULK1) axis is activated in the presence of protective agents, followed by STING dephosphorylation and translocation to the mitochondria. This translocation required tumor necrosis factor receptor-associated factor 2 (TRAF2), an E3 ubiquitin ligase that regulates inflammatory signaling pathways, to bind it and form the STING/TRAF2 complex, causing the ubiquitination and degradation of STING. This series of interactions promoted autophagy and protected against DCM injury 80, 81. Another study examined the relationship between cGAS-STING and obesity-induced cardiomyopathy using HFD-fed db/db mice and H9C2 cells that were exposed to PA stimulation. Their results indicate that mitochondria-derived cytosolic DNA is an essential mediator of cGAS-STING signaling in DCM. In-depth investigations showed that genetic and pharmacologic blockage of STING ameliorated cardiomyocyte damage by decreasing NF-κB-related inflammatory signaling and apoptosis 58. Taken together, these experiments suggest that cGAS-STING activation exacerbates DCM progression.

Sepsis is a life-threatening syndrome that can lead to multiple organ failure. The heart is the most susceptible organ to sepsis, and its damage is always serious and irreversible, which causes related cardiomyopathy and even heart failure (HF) 82-84. Liu *et al.* 84 used lipopolysaccharide (LPS) stimulation to establish *in vivo* and *in vitro* models of sepsis-induced cardiomyopathy (SIC) and to study the mechanism by which mitochondrial aldehyde dehydrogenase 2 (ALDH2) alleviates SIC. They demonstrated that ALDH2's protective effects were mediated by the eradication of the cGAS-STING axis, which was followed by the mitigation of inflammation and apoptosis. These mechanisms decreased cardiac injury markers, including creatine kinase-MB (CK-MB) and LDH, and improved cardiac function. This study suggests that inhibiting cGAS-STING is capable of weakening the effects of SIC. In particular, the STING trafficking augments sepsis-induced cardiac dysfunction by aggravating ROS production, ferroptosis, and inflammation 85. In addition, underlying cGAS-STING-mediated exacerbations of SIC, and the cGAS-STING activation could also fuel the phosphorylation and nuclear translocation of IRF3, resulting in NLRP3 inflammasome formation. Overall, activation of the pathological signaling axis aggravated inflammation, pyroptosis, and apoptosis, thus accelerating cardiac disorder 61. The cGAS-STING activation also contributes to drug-induced cardiomyopathy 79. Doxorubicin (DOX) administration significantly increases cGAS and STING expression levels and downstream effectors, including TBK1, IRF3, and NF-κB p65. Intriguingly, the NLRP3 level is also elevated under this condition. As expected, these changes in molecular expression after the DOX challenge could be partially reversed by silencing STING. This condition can improve myocardial injury and cardiac function dramatically, as shown by decreased activity of myocardial damage markers and increased ejection fractions (EF), fractional shortening (FS). In addition to these apparent changes, other mechanisms may include the inhibition of inflammation and apoptosis.

Hereditary dilated cardiomyopathy is caused by mutations in various genes and is a pervasive, genetically heterogeneous disease that leads to HF. Mutations in the Lamina A/C protein (LMNA) gene, which encodes the nuclear envelope protein Lamin-A/C, are one of the most common abnormalities that lead to dilated cardiomyopathy 86-88. Sirisha M *et al.* 87 found that the expression of cGAS-related signaling molecules such as STING1, TBK1, IRF3, and NF-kB were elevated in LMNA deficient mice compared to control mice. Furthermore, genetic deletion of the cGAS cascade blocked inflammation and apoptosis, increased survival rates, and improved cardiac function. These results support targeting cGAS-STING to treat lamin-related cardiomyopathy.

### 2.3 The Role of cGAS-STING in the ischaemic cardiomyopathy

Ischemic cardiomyopathy is a severe type of CVD that is responsible for most cardiac-related deaths around the world. After myocardial ischemia, the inflammatory response is triggered to promote the repair of cardiomyocytes. However, excessive inflammation can lead to secondary damage to cardiomyocytes. The cells become irreversibly dysfunctional, ultimately culminating in cardiac remodeling and HF 89, 90. For patients going through acute myocardial infarction (AMI), timely and efficient reperfusion treatment can save injured myocardial tissues, while it tends to result in dampening the cardiac damage upon the restoration of blood flow. This phenomenon is called myocardial ischemia-reperfusion (MI/R) injury, which drives a series of reactions such as inflammation, oxidative stress, and cell apoptosis 91. The cGAS-STING pathway also contributes to the pathogenesis of MI and MI/R 6, 12, 91, 92. The instigation of the cGAS-STING pathway contributes to the recruitment of inflammation-related cells like macrophages and the release of chemokines in the infarct area, leading to myocardial necrosis and fibrosis 91, 93. Finally, the cardiac structure and function could be damaged, resulting in heart failure (HF). Since the essential role of cGAS-STING initiation in the MI, the ablation of STING-related molecules protects against MI-induced injuries 8. H-151, a selective antagonist of STING, has been shown to abate inflammation and improve cardiac function after MI, reduce TNF-α, chemokine (C-X-C motif) ligands (CXCL)10, and IL-1β levels, and restore EF and FS. *In vitro* experiments showed that when co-cultured with bone marrow-derived macrophages stimulated by dsDNA, cardiomyocytes treated by H-151 show decreased apoptosis rates and inflammation levels, and their fibroblasts produce less collagen 89. The H-151 is also used to inhibit STING to help repair the infarct areas, infarction-induced myocardial hypertrophy, fibrosis, and cytokine release after reperfusion. In addition, it is reported that cardiomyocyte induced by hypoxia/reoxygenation (H/R) possesses higher expression of cGAS-STING, and either the inhibition of cGAS with RU.521 or suppression of STING with H151 can mitigate the cell apoptosis and cardiac disruption after MI/R, probably via regulation for Bcl‑2/Bax/Caspase‑3 signaling pathways 94. Type 2 diabetes is the risk factor for accelerating the damage of MI/R. It is established that during the development of diabetes, the morphological and functional properties alter, leading to the increased mtDNA leak into the cytosol and activation of the cGAS-STING signaling, exacerbating the MI impacts. The abrogation of cGAS-STING alleviates the inflammation and myocardial damage in diabetic MI/R rats. *In vitro* experiments demonstrated that the inhibition of STING improved the mitochondrial function and cell viability of H9C2 cardiomyocytes challenged by HG and H/R 95. These data imply that obliterating STING can alleviate myocardial damage and improve cardiac function after MI 92.

### 2.4 The role of cGAS-STING in other CVDs

HF is the end-stage of cardiac dysfunction, which is mainly caused by various kinds of cardiogenic factors. The progression of HF is reported to be associated with chronic inflammation 96, and further investigation has identified the crucial role of the cGAS-STING pathway in HF 12, 97. Under the stimulation of pressure overload, the cardiomyocytes may undergo mitochondrial injury, followed by the mtDNA leakage to the cytosol and the activation of the cGAS-STING axis. Then, the undue inflammation cascade and cell apoptosis occurs, driving the hypertrophy and fibrosis of cardiomyocytes. However, the genetic ablation of cGAS in mice challenged by transverse aortic constriction blocked the early inflammatory response and apoptosis, alleviating cardiac remodeling and restoring cardiac functions 97. Additionally, the cGAS-STING signaling pathway is involved in the development of myocarditis. In mice treated with aPD-1 therapy to induce immune checkpoint inhibitor-related myocarditis, activated GSDME mediates pyroptosis, leading to mitochondrial injury and subsequent release of mtDNA. This release activates the cGAS-STING pathway, which exacerbates myocardial inflammation. In contrast, genetically blocking STING in mice results in lower levels of markers associated with myocardial damage, reduced T-cell infiltration, and improved cardiac function. These beneficial effects are comparable to those achieved by the ablation of gasdermin E (GSDME) 98. Similarly, the administration of X6 in a mouse model of autoimmune myocarditis effectively inhibited the release of cGAMP and the subsequent cGAS-STING-induced production of IFN-β and expression of ISGs. This intervention ultimately reduced endocardial inflammation and fibrosis. These findings suggest that targeting the cGAS-STING pathway could alleviate the progression of HF and myocarditis.

## 3. The PERK-eIF2α in CVDs

### 3.1 The PERK-eIF2α pathway in different cellular phenotypes

**Figure 2:**
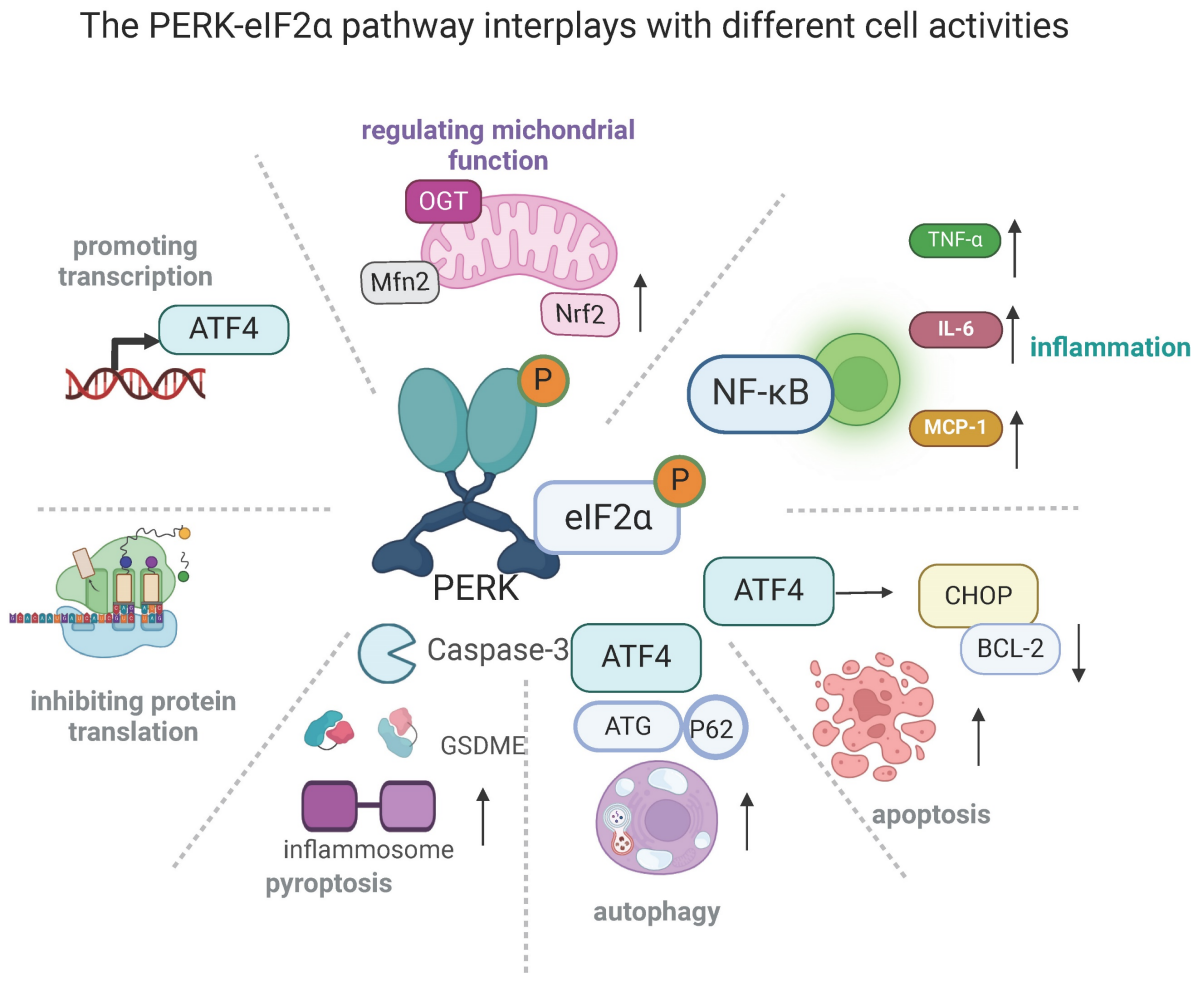
The PERK-eIF2α pathway connects to different cellular activities (created with BioRender.com). The PERK-eIF2α pathway plays a master role in several critical cellular functions. Its fundamental effects are the selective transcription of genes such as ATF4 and the arrest of protein translation. Excessive ATF4 activation can promote cell apoptosis. It can also induce ER stress-related inflammation by increasing the translocation of NF-κB, thus increasing the release of tumor necrosis factor-α (TNF-α), interleukin (IL)-6, and monocyte chemoattractant protein-1 (MCP-1). Additionally, it triggers pyroptosis via gasdermin E (GSDME). PERK-eIF2α activation can positively regulate autophagy by up-regulating autophagy related 5 (ATG5) and ATG7, as well as involving ATG12 and p62. Finally, its activation stabilizes mitochondrial functions in several ways: it promotes mitochondrial hyperfusion via the PERK-eIF2α-ATF4 pathway, it increases the formation of mitochondrial cristae via the PERK-O-linked N-acetyl-glucosamine transferase pathway, it facilitates mitophagy through the PERK-transcription factor EB (TFEB) pathway, and it favors oxidative phosphorylation via the PERK-nuclear factor erythroid 2-related factor 2 (Nrf2) pathway.

The PERK-eIF2α mediated pathway plays a major role in several cellular disorders, including inflammation, apoptosis, autophagy, and pyroptosis (**Figure 2**) 99. Inhibiting PERK-eIF2α has been shown to reduce ER stress-related inflammation by preventing the translocation of NF-κB and decreasing the release of tumor necrosis factor-α (TNF-α), IL-6, and monocyte chemoattractant protein-1 (MCP-1) 100. Notably, PERK-eIF2α downregulation alleviated the inflammation and apoptosis of cardiomyocytes that occurs during the development of ischemic stroke and thus protected against resultant injury 99, 101. Apoptosis was also blunted by the deacetylation of PERK-regulated eIF2α, which is responsible for the amelioration of cardiomyocyte death and cardiac function 102. Additionally, the levels of several ER-stress-related molecules, including p-PERK, p-eIF2α, ATF4, and CHOP, were up-regulated in alveolar type II epithelial cells after perfluorooctane sulfonate treatment. However, the use of a PERK inhibitor partially attenuated GSDME-triggered pyroptosis and inflammatory factor secretion, thereby mitigating lung injury 103. There is a significant relationship between ER stress and autophagy. It is established that activating PERK-eIF2α increased rates of autophagy by up-regulating ATG5 and ATG7 104. Additionally, ATG12 and p62 were confirmed to be involved in this process 105. Notably, autophagy negatively regulates the PERK-eIF2α pathway, which also participates in the interplay between apoptosis and autophagy. By targeting PERK-eIF2α mediated pathways, the neurotoxins were shown to instigate both autophagy and apoptosis in neurons 106. Further studies using a related inhibitor verified that activating autophagy was protective against cellular injury due to the alleviation of apoptosis. Activation of PERK-eIF2α can lead to favorable effects, such as regulating mitochondrial functions. Previous studies have shown that PERK located in MAMs physically interacts with Mitofusin‑2 (Mfn2) and regulates mitochondrial morphology, calcium (Ca^2+^) homeostasis, and oxidative stress 16, 107-109. The PERK-eIF2α pathway also stabilizes mitochondrial function in a multitude of ways, including promoting mitochondrial hyperfusion via the PERK-eIF2α-ATF4 pathway, increasing the production of mitochondrial cristae via the PERK-O-linked N-acetyl-glucosamine transferase pathway 110, facilitating mitophagy through the PERK-transcription factor EB (TFEB) pathway 16, 111, 112, and increasing oxidative phosphorylation via the PERK-nuclear factor erythroid 2-related factor 2 (Nrf2) pathway 43. Thus, PERK-eIF2α is closely involved in diverse signaling transduction and plays a key role in numerous normal and aberrant mitochondrial functions.

### 3.2 The role of PERK-eIF2α in AS

Oxidized low-density lipoprotein (ox-LDL) is a crucial component in the development of AS. As cholesterol continues to accumulate within a vascular lesion, smooth muscle cells (SMCs) switch phenotype in a process that is characterized by the downregulation of SMC differentiation genes and the upregulation of macrophage or fibroblast-related genes. This process accelerates the formation of atherosclerotic plaques. Previous studies have shown that the p-PERK and p-eIF2α levels are higher in the ApoE-/- mice 113. In addition, the influx of cholesterol into cardiomyocytes facilitates the UPR in the ER, thus activating PERK, IRE 1α, and ATF. This cascade can also promote the modulation of SMC-related phenotypic conversion, leading to an increase in plaque loads 42. Pericentrin is a key component of the microtubule organizing center, which enables the accurate completion of cellular division. Pericentrin deficiency in SMCs can actuate heat shock factor 1 (HSF1), and consequently augment the viability of HMG-CoA reductase (HMGCR), thus increasing cholesterol biosynthesis. The increases in cholesterol biosynthesis induced both PERK signaling and the phenotypic modulation of SMCs, which in turn increased atherosclerotic plaque burdens 13, 114. However, blocking PERK decreased the expression of several macrophage- and UPR-related molecules, such as phosphorylated eIF2α and ATF4, thereby alleviating AS progression 115. Vascular calcification (VC), particularly intimal VC, is a risk factor for the development of vulnerable atherosclerotic plaques 116, 117. Intriguingly, the expression of ER-related molecules such as PERK, eIF2α, ATF4, GRP78, and CHOP was found to be significantly higher in VC rat models compared to wild-type rats. Allicin administration, however, suppressed PERK activation and relieved aortic VC. Further study identified that the pharmacological inhibition of eIF2α phosphorylation helped ameliorate VC in the aorta 116, 118. The above findings indicate that the activation of the PERK-eIF2α plays an essential role in the process of AS, and the blockage of PERK may dampen the advancement of AS burden.

### 3.3 The role of PERK-eIF2α in cardiomyopathy and HF

Accumulating evidence suggests that PERK-eIF2α plays a major role in the development of cardiomyopathy, although some disagreement persists 17, 119-121. The PERK's involvement in dilated cardiomyopathy has been well-documented 122. For instance, melatonin alleviated dilated cardiomyopathy by targeting the PERK-eIF2α pathway. To be exact, melatonin mitigated the tether between PERK and GRP78, thereby promoting PERK's dissociation and phosphorylation. The activated PERK then mitigated myocardial injury and improved cardiac function by up-regulating autophagy pathways. In-depth investigations demonstrated that inhibiting PERK weakened the protective effects of melatonin in dilated cardiomyopathy 17. Paradoxically, it is documented that the lncRNA H19 downregulated the expression of p-PERK and CHOP, which then ameliorated cardiomyocyte apoptosis and fibrosis, and remarkably improved cardiac function in DCM mice 123. Likewise, LCZ696 was found to protect against DCM-induced inflammation, ER Stress, and apoptosis by inhibiting advanced glycation end-products (AGEs)/NF-κB and PERK-eIF2α directed cascades, thus improving glucose metabolism, lipid metabolism, and myocardial injury 119. *In vitro* experiments, inhibiting PERK and its downstream molecules decreased apoptosis and ER stress. 120.

GCN2 is an enzyme that phosphorylates PERK, and its deficiency is observed to be protective in DCM 124. Loss of GCN2 could mitigate cardiac contractile disruption, myocardial fibrosis, apoptosis, and oxidative stress in DOX-induced cardiomyopathy by attenuating eIF2α-CHOP, and knockdown of eIF2α in the DOX-challenged H9C2 cells resulted in the higher survival rates and decreased oxidative stress 125. Aiming to understand the ER stress effects in Lamin-A/C-variant-induced dilated cardiomyopathy, Pietrafesa *et al.* 126 established an *in vitro* model and discovered that levels of PERK and its downstream signal molecules were elevated in the context of this mutation. Additionally, inhibiting PERK promoted myocardial survival, probably by increasing levels of phosphorylated A serine/threonine-specific protein kinase B (AKT). Phospholamban is a crucial modulator of cardiac calcium balance and contractility, and arginine deficiency at position 14 is associated with both dilated cardiomyopathy and ventricular arrhythmias. Surprisingly, the protective effects of PERK have been validated in cardiomyopathy by utilizing PERK knockdown in human induced pluripotent stem cells (hiPSC-CMs) to establish an R14del model 127. Concerning other types of cardiomyopathies, PERK-eIF2α mediated ER stress is also found to be involved in the protective effects of hydrogen sulfide against SIC 128. These findings may imply that the PERK-eIF2α participated in the pathophysiology of cardiomyopathy via regulation of cell death, inflammation, fibrosis, and metabolism. HF is the final stage of various cardiomyopathies 129, 130. To investigate the mechanism underlying dapagliflozin's protective effects in HF, Ren *et al.* 131 created a transverse aortic constriction (TAC) mouse model, as well as angiotensin (Ang) II-induced cardiomyocytes. They determined that the expression of p-PERK-p-eIF2α-ATF4-CHOP was increased in model groups. However, dapagliflozin administration improved cardiac function and reduced myocardial hypertrophy and fibrosis. Cardiomyocyte apoptosis also decreased, as exhibited by increases in pro-apoptotic markers such as caspase 3 and Bax-1 and decreases in TUNEL-stained cells. Several protective effects were also decreased after the use of ER stress inducers. These results indicate that inhibiting PERK-eIF2α-directed apoptosis helped alleviate myocardial remodeling during the development of HF. Additionally, this inhibition was achieved in a Sirtuin 1 (SIRT1)-dependent way, which also suppressed cardiomyocyte apoptosis in DCM via the PERK-eIF2α pathway 132. Activating PERK, however, was also verified to help alleviate hypertrophic remodeling 133. It is therefore feasible that PERK may have divergent effects on cardiac disease depending on the context and signaling involved.

### 3.4 PERK-eIF2α's role in MI injury

Myocardial reperfusion injury primarily derives from excessive oxidative stress, increased ER stress, and/or cardiomyocyte apoptosis 134-137. Consistently, in both cellular H/R models and animal myocardial I/R models, Perk/eIF2α/ATF4 signaling pathways were highly activated compared with control groups 134. Anaerobic exercise exerts protective effects in I/R mice, which were in part mediated by the inactivation of the PERK/eIF2α/ATF4 pathway. Mechanistically, anaerobic exercise attenuated apoptosis and mitophagy by downregulating the PERK/eIF2α/ATF4 pathway, which consequently reduced myocardial damage and improved cardiac function. Furthermore, the PERK takes action in the pathological development of certain arrhythmias. Liu *et al.* 138 found that both genetic and pharmacological inhibition of PERK shortened Q and T waves on ECGs (thus shortening QTc intervals), decreased ventricular tachycardia episodes, and heightened survival rates after MI, suggesting that inhibiting PERK may reduce arrhythmia risk after MI.

## 4. The Implication of the cGAS-STING/PERK-eIF2α pathway in CVDs

As discussed, there is a significant connection between the cGAS-STING and PERK-eIF2α pathways. Evidence suggests that inhibiting either pathway can alleviate the pathological progression of various CVDs, including atherosclerosis, myocardial ischemia-reperfusion injury, and cardiomyopathy, by reducing inflammation, apoptosis, macrophage accumulation, and smooth muscle cell phenotype switching. While both pathways have been extensively studied individually, few investigations have explored their combined effects. Therefore, research targeting the cGAS-STING/PERK-eIF2α pathway may uncover novel therapeutic targets for CVDs. Notably, this combined pathway has been shown to mitigate senescence and organ fibrosis, which are critical mechanisms in the development of CVDs 139, 140. The STING-p-PERK-p-eIF2α-ATF4 pathway has also consistently been verified to be involved in inflammation and collagen deposition and synthesis while attenuating the cGAS-STING/PERK pathway reduces fibroblast activation and extracellular matrix production 141, 142. Additionally, aiming to explore the association between ER stress and cGAS-STING in cardiac pathophysiology, Zhang *et al.* 143 first demonstrated greater p-PERK and p-eIF2α expression in patients with DCM and hypertrophic cardiomyopathy (HCM). Then they showed that knockout of STING resulted in the elimination of p-PERK and p-eIF2α in mice with aortic binding. Likewise, the suppression of PERK reduced STING expression. Blocking the cGAS-STING/PERK-eIF2α pathway alleviated cardiomyocyte hypertrophy, inflammation, and fibrosis, and thus attenuated myocardial damage. With regards to AS, p-STING and p-PERK expression were increased in patients with AS as well as in ox-LDL-treated endothelium. The knockdown of STING alleviated endothelial injury, inflammatory responses, and atherosclerotic plaque areas. The intrinsic mechanism was explored using chromatin immunoprecipitation sequencing, and results confirmed that, during the development of AS, activated STING recruits PERK, leading to the recruitment of transcription factors such as IRF3 and NF-kB. Simultaneously, the activated STING/PERK complex promotes interactions between IRF3, NF-kB, and bromodomain protein 4 (BRD4), which form a super-enhancer and further direct the transcription of inflammatory genes 7. Taken together, it is conceivable to extrapolate that the cGAS-STING/PERK-eIF2α interaction plays a vital role in an array of CVDs. Targeting this pathway may be an exciting new approach to the treatment of CVDs.

## 5. Inhibition of the cGAS-STING/PERK-eIF2α in CVDs and potential clinical value

With a growing body of research highlighting the significance of the STING/PERK pathway in numerous disorders, our understanding of the inhibitors of this pathway has also improved. The common inhibitors in the application of pre-clinical experiments about CVDs are listed in **Table 3**. For STING, mature antagonists include H-151, C-176, and C-178, which block palmitoylation at cysteines 88 and 91 (C88/91) and thus block STING's activation 144. C-176 and C-178 are nitrofuran derivatives that covalently bind with one of STING's nucleophilic side chains via their 4-position furan rings, followed by rearrangement of STING and blocking of STING palmitoylation. C-176 and C-178 are only able to react with mice STING, but H-151 can block human STING activation with high specificity 145. Common inhibitors of cGAS include RU.521, G108, G150, and PF-06928215. These inhibitors bind with cGAS' active site with high affinity and inhibit the production of cGAMP 146. Although RU.521 only reacts with murine cGAS, G108, and G150 can also combine with human cGAS 147. PF-06928215 has a high affinity with cGAS residue to impede its activation, although it showed no activity in cellular cGAS assays assessing dsDNA-induced IFN expression. The alkyl chain of PF-06928215 interplay with a small hydrophobic pocket of cGAS formed between Tyr436 and His437 148. In addition, several natural products have been discovered to prohibit cGAS-STING-induced pathways, including Astin C, flavonoids, and Scutellarin 91, 94. GSK2656157, GSK2606414, and AMG44 are known to negate PERK by occupying its ATP-binding site, which can inhibit PERK in the nanomolar range 149. However, the potent inhibition of phospho-PERK and histologic changes showed no effects on the PERK-mediated classical signaling, since the improper concentration of it may induce other related enzymes that promote this cascade. Integrated stress response inhibitor (ISRIB) can be used to inhibit eIF-2α's activation 150, 151. However, GSK2656157 specifically has no effects on eIF2α 152. Considering the significant crosstalk between the cGAS-STING and PERK-eIF2α pathways, a combination of drugs targeting both STING and PERK or certain compounds that inhibit both STING and PERK may have better effects in certain CVDs.

As discussed in this article, the individual and collaborative roles of the cGAS-STING and PERK-eIF2α pathways are crucial in the progression of CVDs. Understanding these pathways opens new avenues for clinical strategies in the diagnosis, treatment, and prognosis of patients.

Firstly, in addition to traditional diagnostic methods such as measuring myocardial enzymes and conducting echocardiograms, advanced technologies can be employed to assess the concentrations of cGAS-STING, PERK-eIF2α, and related downstream molecules, including IFN, IL, MCP-1, ROS, and ATF. This assessment can help gauge disease severity and facilitate risk stratification among patients. A more comprehensive understanding of a patient's condition before reperfusion surgery could enable healthcare providers to develop more precise therapeutic approaches.

Secondly, many current clinical treatments are insufficient due to myocardial remodeling and fibrosis, and they may cause adverse effects such as severe hypotension, edema, liver and kidney dysfunction, or hemorrhage, leading to patient intolerance. With an increasing number of preclinical studies investigating inhibitors of the cGAS-STING and PERK-eIF2α pathways, there is a promising opportunity to introduce these therapies into clinical practice. Additionally, considering the interaction between STING and PERK, combining inhibitors of both pathways may enhance efficacy while maintaining safety.

Previously, STING inhibitors primarily targeted the classical cGAS-STING pathway, neglecting the STING-PERK axis. Furthermore, research on PERK antagonists remains scarce, highlighting the urgent need to explore novel compounds or natural products that can simultaneously target both STING and PERK, such as compounds centered on the motif-helix aa322-343 of STING.

Lastly, the application of these inhibitors could provide alternative therapies for patients who do not achieve optimal results with existing treatments or experience severe side effects. A more thorough evaluation of disease status coupled with personalized treatment plans could lead to better prognoses for patients. From a clinical perspective, targeting the cGAS-STING/PERK-eIF2α pathway may represent a valuable therapeutic strategy for alleviating CVDs.

## 6. The nanomedicine for the treatment of CVDs by targeting cGAS-STING/PERK-eIF2α pathway

Investigating compounds within the cGAS-STING/PERK-eIF2α pathway holds significant clinical promise. However, many pharmacological experiments have struggled due to poor local drug activity and systemic toxicity arising from a lack of selectivity for target cells. This challenge has fostered advancements in biomaterials to address these issues, particularly through the use of nanomaterials, which have gained increasing attention.

The application of nanomaterial technology in inhibiting the cGAS-STING pathway is on the rise. Unlike conventional biomedical materials, nanomaterials exhibit enhanced properties, such as safety and biocompatibility, making them effective carriers for delivering cGAS or STING inhibitors to modulate the cGAS-STING signaling pathway 153, 154. Research has shown that nanomedicine can have beneficial effects in treating various CVDs by primarily suppressing inflammation and immune responses 155-157. For example, a biomimetic hydrogel system, Rb1/PDA-hydrogel, composed of ginsenoside Rb1 and polydopamine nanoparticles loaded into carboxylated chitosan, has been reported to improve outcomes in myocardial infarction treatment. This system reduces myocardial fibrosis, increases ventricular wall thickness, and enhances cardiac function by disrupting the crosstalk between mtDNA and cGAS, thereby inhibiting inflammation and macrophage polarization. Notably, this biomaterial demonstrates excellent adhesion to myocardial surfaces, making it suitable for deep myocardial injection due to its wet adhesion properties and conductivity 154. While research on nanomedicine targeting PERK inhibition is limited, one study indicated that delivering a PERK inhibitor via biomimetic nanoclusters significantly improved cardiac function and inhibited aneurysmal dilatation. Remarkably, weekly injections of this targeted PERK inhibitor prevented the progression of pre-existing aneurysmal lesions 158. Additionally, cysteine-based nanoparticles have been utilized to encapsulate the antifibrotic drug PF543, effectively delivering it to activated cardiac fibroblasts within the infarct area. This approach not only ensures prolonged drug release but also markedly reduces kidney and liver toxicity compared to traditional systemic therapies 155. Overall, nanomaterials have shown great potential in treating CVDs by efficiently inhibiting inflammation and immune responses. Given the crucial role of macrophages in these processes, recent research has focused on macrophage-targeted nanomedicine, although challenges remain 159. Future biomedical materials, especially nanomaterials, could be designed to specifically target cells involved in CVDs, such as macrophages and fibroblasts, to effectively inhibit macrophage polarization, inflammation, and fibrosis, ultimately alleviating conditions like atherosclerosis and myocardial infarction. In summary, the promising field of nanomedicine presents new possibilities for targeted therapies in the treatment of CVDs.

## 7. Conclusion and Limitations

In our review, we explored the critical roles of the cGAS-STING and PERK-eIF2α pathways in CVDs and hypothesized that these pathways may interact synergistically to accelerate disease progression. These insights could provide a new perspective on the cGAS-STING/PERK-eIF2α pathway, potentially enhancing the precision of targeted therapies for CVDs. With the rise of nanomedicine, the prospects for effectively inhibiting the cGAS-STING/PERK-eIF2α pathways appear increasingly promising. However, the more specific mechanisms underlying the association between STING and PERK remain unclear, and there is ongoing debate about how they regulate each other. Comprehensive investigations into these exact mechanisms are needed and could help transform the present basic research results into clinical practice. Additionally, due to the complexity of the crosstalk, activating this pathway can occasionally exert opposite effects depending on the context of CVDs, posing a challenge in determining whether to administer antagonists to patients with similar conditions. Therefore, we still need to determine the precise cardiac disorders within which PERK is activated and causes ensuing damage, as well as the cardiac disorders where PERK activation does not cause damage. Additionally, despite the evidence demonstrating the suppression of the cGAS-STING/PERK-eIF2α pathway *in vivo* and *in vitro* settings, there has been a lack of adequate implementation in clinical practice. The potential side effects of combining inhibitors targeting both pathways have yet to be thoroughly investigated. Additionally, compounds capable of simultaneously inhibiting both the cGAS-STING and PERK-eIF2α pathways remain largely unexplored. While promising advancements in nanomedicine have facilitated the inhibition of STING and PERK, several factors may hinder the widespread adoption of nanomaterials. For instance, their small size and enhanced penetration can increase the risk of toxicity to vital organs, such as the heart and brain. Furthermore, the release of nanomaterials into the environment could pose ecological risks 160, 161. Therefore, further research is needed to establish appropriate dose ranges and to explore additional methods for assessing the toxicity of nanomaterials, including the use of various 3D models and experimental approaches. Additionally, the metabolic pathways of most nanoparticles in the body remain largely unexplored 162. In other words, we need to develop advanced tracking modalities, such as radioactive tracing and fluorescence bioimaging, to accurately locate nanoparticles. Additionally, achieving quality control for nanoformulations requires sophisticated technology and a quality-by-design approach, along with multidisciplinary collaboration. Given both the potential benefits and uncertainties, the inhibition of the cGAS-STING/PERK-eIF2α pathway for treatment warrants ongoing and in-depth exploration in the future.

## Figures and Tables

**Table 1:** The cGAS-STING pathway in CVDs

Model	Targets	Inhibitors	Activators	Cell phenotype	Related molecules	Results	Reference
HUVECs	cGAS, STING	shRNA-cGAS	CSE	apoptosis, inflammation	mtDNA	CSE induced endothelial dysfunction and aggravated AS progression via activation of cGAS-STING signaling	9
ApoE-/-mice, oxLDL-treated macrophages and PBMCs	mitochondrial DNA	shRNA-cGAS	TDP43	inflammation, lipid uptake of macrophages	mtDNA, NF-κB	TDP43 promoted inflammation and foam cell formation, augmenting AS progression through triggering mitochondrial DNA release to activate cGAS-STING signaling	75
HFD-fed ApoE-/-mice and Ldlr-/-mice, HUVECs treated with PA	cGAS, STING	RU.521 C-176 siRNA-cGAS siRNA-STING	IQGAP1	pyroptosis, oxidative stress	NLRP3	IQGAP1 induced mitochondrial damage and activation of cGAS-STING pathway during the development of AS	5
CKD/ApoE-/- mice, human VSMC and Mice VSMC	cGAS, STING	knockout of cGAS and STING, C-176		premature, senescence and phenotypic switching of VSMC	mtDNA, IFN-1	The activation of cGAS-STING decreased content of VSMC and fibrous cap, alleviated plaque area and increased plaque stability	10
HFD-fed mice	STING	shRNA-STING	diABZI	inflammation, pyroptosis	NLRP3	The inhibition of STING improved HFD-induced cardiac function and mitigated myocardial fibrosis	76
HFD-fed db/db mice, PA-induced H9C2 cells	cGAS, STING	siRNA-STING, C-176	extracted mtDNA	inflammation, apoptosis	mtDNA, IRF3, NF-κB	The inhibition of STING improved cardiac function and mitigated myocardial fibrosis in DCM	58
HFD induced mice, HG and PA-induced H9c2 cells	STING	C-176	diABZI	pyroptosis, apoptosis	MITOL, NLRP3, GSDMD	MITOL activation ameliorated DCM- induced myocardial injury and cardiac disorder by inhibiting cGAS/STING signaling	78
STZ-induced (T1D) mice and (db/db) (T2D) mice, HG-induced neonatal rat cardiomyocytes	STING	Metrnl	siRNA- Metrnl	Apoptosis, autophagy	LKB1, AMPK, ULK1	Metrnl ameliorates DCM injury and cardiac function via inactivation of cGAS/STING signaling	80
LPS-induced mice and H9C2 cells	cGAS	siRNA-cGAS ALDH2		inflammation, and apoptosis, oxidative stress	ALDH2	ALDH2 alleviated LPS-induced cardiac dysfunction, and protected against SIC-induced injury via inhibition of cGAS/STING pathway	84
LPS-induced mice, H9C2 cells and macrophages	STING		ICA69	inflammation, ferroptosis, oxidative stress	ICA69	ICA69 deficiency alleviated septic cardiac dysfunction and damage via upregulation of STING mediated pathway	85
LPS-induced mice NRCMs, and H9C2 cells	STING	knockout of STING, siRNA-STING		inflammation, apoptosis, pyroptosis	NLRP3, IRF3	Deficiency of STING alleviated SIC-triggered cardiac function and injury	61
Dox-induced mice and HL-1 cardiomyocytes	STING	siRNA-STING		inflammation, apoptosis	NLRP3	The knockdown of STING improved survival and cardiac function of DOX-treated mice and alleviated cell injury	79
Mice model of LMNA-related cardiomyopathy	cGAS, STING	knockout of cGAS and STING		nuclear envelope rupture		cGAS or STING deletion does not rescue dilated cardiomyopathy in LMNA-CKO mice	82
Lmna F/F mice	cGAS	knockout of cGAS		inflammation, apoptosis	TBK1, IRF3, and NF-kB	The ablation of cGAS prolonged survival, improved cardiac function, and attenuated myocardial fibrosis in the LMNA-deficient mice	87
MI mice, cardiac cardiomyocyte and fibroblasts after co-culturing them with the supernatant of stimulated BMDMs	cGAS, STING	H-151	DMXAA	inflammation, apoptosis	TBK1, IRF3	The inhibition of cGAS/STING mediated pathway alleviated cardiac function and myocardial fibrosis	89
MI model	STING	H-151		inflammation		The STING blockage decreased infarction size and scarring, relieved cardiac function and reduced myocardial fibrosis and hypertrophy after reperfused MI	92
Mice challenged by TAC, neonatal rat ventricular myocytes	cGAS, STING	knockout of STING, shRNA-cGAS, shRNA-STING	iNOS, compound 3	inflammation, macrophage polarization	mtDNA	The inhibition of cGAS/STING alleviated the myocardial fibrosis and cardiac function, and mitigated the pressure- overload induced myocardial injury	93
I/R mice model, H9c2 cells exposed to H/R	cGAS, STING	RU.521 H-151		apoptosis	Bcl‑2/Bax/ Caspase‑3	Scutellarin ameliorated the cardiac function and infarct area, decreasing the I/R injury	94
Diabetic mice with I/R, H9C2 cells challenged by HG and PA	STING	H-151	decreased mitofusins 2	inflammation	mtDNA	The inhibition of cGAS/STING improved the cardiac function and alleviated the diabetic I/R injury	95
Mice chanllenged by TAC-induced HF	cGAS	sh-cGAS		Inflammation, apoptosis		The inhibition of cGAS relieved the cardiac function and remodeling, mitigating HF induced by pressure-overload	97
mice model with aPD-1-induced myocarditis	STING	STING mutant	Gasdermin-E	Pyroptosis, inflammation, T cell infiltration	mtDNA	The knockout of Gasdermin-E improved the myocardial morphology, and cardiac function, mitigating myocarditis	98

HUVECs: Human umbilical vein endothelial cells; CSE: cigarette smoke; mtDNA: mitochondrial DNA; TDP43: transactive response DNA-binding protein∼43kDa; PBMCs: peripheral blood mononuclear cells; HFD: high-fat diet; IQGAP1: IQ motif-containing GTPase-activating protein; NLRP3: NOD-like receptor protein 3; IRF3: interferon regulatory factor 3; NF-κB: nuclear factor-kappa B; DCM: diabetic cardiomyopathy; PA: palmitic acid; HG: high-glucose; MITOL: mitochondrial ubiquitin ligase; GSDMD: gasdermin D; Dox: doxorubicin; STZ: streptozotocin; LKB1: liver kinase B1; AMPK: adenosine monophosphate activated protein kinase; ULK1: Unc-51 like autophagy activating kinase 1; ALDH2: mitochondrial aldehyde dehydrogenase 2; SIC: sepsis-induced cardiomyopathy; ICA69: Islet cell autoantigen 69; NRCMs: neonatal rat cardiomyocytes; MI: myocardial infraction; BMDMs: bone marrow-derived macrophages; DMXAA: 5, 6-dimethylxanthenone-4-acetic acid; TBK1: TANK-binding kinase 1; TAC: transverse aortic constriction; iNOS: inducible NO synthase; I/R: ischemia/reperfusion; H/R: hypoxia/reoxygenation; HF: heart failure.

**Table 2:** The PERK-eIF2α pathway in CVDs

Model	Targets	Inhibitors	Activators	Cell phenotype	Related molecules	Results	Reference
ApoE-/-mice, oxLDL- induced HUVECs	PERK, eIF2α	atorvastatin			AMPK	Atorvastatin reduced the level of p-PERK and p-eIF2α, alleviating AS plaque formation and increased plaque stability	113
Cholesterol-loaded VSMCs	PERK, eIF2α	4-PBA, ISRIB, shRNA-PERK		phenotypic switch of SMCs	ATF4	The inhibition of PERK decreased plaque burden and AS progression	42
Hyperlipidemic mice, Pcnt^SMC-/-^SMCs	PERK, eIF2α	ISRIB	pericentrin deficiency	phenotypic modulation of SMCs	HSF-1	Pericentrin deficiency augmented cholesterol biosynthesis and AS plaque burden through PERK activation	13
Hyperlipidemic mice, VSMCs induced by free cholesterol	PERK	knockout of PERK		phenotypic modulation of SMCs	none	The inhibiton of PERK blocked AS plaque formation	115
Rat administrated by vitamin D3	PERK, eIF2α	ISRIB		calcium deposition and ALP activity	ATF-4	The inhibition of eIF2α ameliorated the pathogenesis of vascular calcification	116
Rat administrated by vitamin D3	PERK, eIF2α	allicin		calcium deposition and ALP activity osteoblastic differentiation of VSMCs	ATF-4	Allicin decreased the level of p-PERK and p-eIF2α, ameliorating the vascular calcification and stiffness	118
T1DM induced cardiomyopathy mice, high glucose-treated neonatal rat ventricular myocytes	PERK, eIF2α	GSK2656157	Melatonin	autophagy	ATF-4 GRP78	The activation of PERK improved cardiac function and ameliorated myocardial injury in DCM	17
DCM mice induced by STZ	PERK, eIF2α	LCZ696		inflammation, apoptosis	CHOP	LCZ696 alleviated lipid and glucose metabolism, protected against DCM-Induced myocardial injury through inhibiting AGEs/NF-κB and PERK/CHOP	119
DCM mice induced by STZ, HL-1 cardiomyocyte stimulated by high-glucose	PERK	LncRNA H19	thapsigargin	apoptosis		H19 improved left ventricular dysfunction and decreased fibrosis in DCM	123
DCM mice induced by STZ and HFD, H9C2 cardiomyocyte induced by high glucose or palmitic acid	eIF2α	GCN2 deficiency		inflammation, apoptosis	CHOP, ATF4	GCN2 deficiency ameliorated cardiac dysfunction and remodeling in DCM by reducing fibrosis, lipo-toxicity and oxidative stress	124
Mice induced by DOX	eIF2α	GCN2 deficiency		apoptosis	CHOP, ATF4	GCN2 deficiency improved myocardial contractile function and fibrosis in DOX-induced cardiomyopathy	125
Rats administrated by CLP	PERK, eIF2α	Hydrogen Sulfide		apoptosis	CHOP	Hydrogen sulfide ameliorated myocardial injury caused by sepsis via suppressing PERK-eIF2α related ER stress	128
LMNA R321X-cardiomyocytes	PERK, eIF2α	empaglifozin		apoptosis	CHOP	Empaglifozin attenuated LMNA cardiomyopathy via inhibition of PERK	126
TAC induced mice, Ang II-induced cardiomyocyte	PERK, eIF2α	Dapagliflozin		migration and transformation of fibroblast, apoptosis	STRT1	Dapagliflozin attenuated pressure overload-induced myocardial hyper trophy and remodeling via inhibition of PERK- eIF2α	131
I/R rats, H/R-indeced H9c2 cardiomyocyte	PERK, eIF2α	M_2_AChR		mitophagy and apoptosis	ATF-4	M_2_AChR activation resisted I/R-induced myocardial injury and improving cardiac function via inhibition of PERK- eIF2α	134
MIRI rats, H/R-indeced H9c2 cardiomyocyte	PERK, eIF2α	cFLIP_L_		apoptosis	p38 MAPK	cFLIP_L_ attenuated activation of PERK, and reduced myocardial infarction and increased the viability of H9c2 cells	135
DM+I/R rat, H9C2 cardiomyocyte treated with HG and H/R		Ferrostatin-1, Salubrinal,		ferroptosis	ATF4	Inhibition of ERS inhibited ferroptosis and ameliorated diabetes MIRI	136
MI mice, isolated cardiomyocytes from remote zone of the left ventricle scar	PERK	knockout of PERK, GSK		ion channel activation		Inhibition of PERK reduces arrhythmia risk after MI	138

AS: atherosclerosis; HUVECs: human umbilical vein endothelial cells; AMPK: adenosine monophosphate-activated protein kinase; VSMCs: vascular smooth cells; 4-PBA: 4-phenylbutyric acid; ISRIB: integrated stress response inhibitor; HSF-1: heat shock factor 1; Pcnt: Pericentrin; ATF4: activating transcription factor 4; ALP: alkaline phosphatase; T1DM: type 1 diabetes mellitus; GRP78: Glucose-regulated protein 78; STZ: streptozotocin; AGEs: advanced glycation end-products; CHOP: C/EBP homologous protein; GCN2: general control nonderepressible 2; DOX: doxorubicin; Lamina A/C protein; CHOP: CCAAT-enhancer-binding protein homologous protein; TAC: transverse aortic constriction; M_2_AChR:M2 Acetylcholine receptor; I/R:ischemia-reperfusion; H/R: hypoxia/reoxygenation; MIRI: myocardial ischemia-reperfusion injury; CLP: cecal ligation and puncture; ERS: endoplasmic reticulum stress.

**Table 3:** The inhibitors of cGAS-STING/PERK-eIF2α in CVDs

Inhibitor	Effects in CVDs	Reference
RU.521 C-176	Alleviated mitochondrial damage and activation of cGAS-STING pathway during the development of AS	5
C-176	Increased content of VSMC and fibrous cap, alleviated plaque area and increased plaque stability	10
C-176	Improved cardiac function and mitigated myocardial fibrosis in DCM	49
H-151	Alleviated cardiac function and myocardial fibrosis in MI mice	62
H-151	Decreased infarction size and scarring, relieved cardiac function and reduced myocardial fibrosis and hypertrophy in reperfused MI mice	60
RU.521 H-151	Decreased cell apoptosis and alleviated cardiac function in MI/R mice	94
H-151	Alleviated the LDH release and infarction area, increased cell viability and cardiac function, mitigated the MI/R injury in mice	95
ISRIB	Decreased plaque burden and AS progression	65
ISRIB	Reduced cholesterol biosynthesis and AS plaque burden	13
ISRIB	Ameliorated the calcium deposition and pathogenesis of vascular calcification	104
GSK2656157	Promoted cardiac disfunction and myocardial injury in DCM	17
GSK2606414	Reduces arrhythmia risk after MI	126

AS: atherosclerosis; VSMC: vascular smooth muscle cell; DCM: diabetic cardiomyopathy; MI: myocardial infarction; MIR: myocardial ischemia/reperfusion

## Abbreviations

cGAS: the cyclic 2′3′ GMP-AMP (cGAMP) synthase
cGAMP: cyclic guanosine monophosphate adenosine monophosphate
STING: stimulator of interferon genes
IFN: type I interferon
TBK1: TANK-binding kinase 1
IRF3: IFN regulatory factor 3
IL: interleukin
PERK: protein kinase RNA-like ER kinase
eIF2α: eukaryotic initiation factor 2α
UPR: unfolded protein response
CVDs: cardiovascular diseases
GRP78: glucose-regulated protein
ATF4: activating transcription factor 4
ISGs: type I interferon (IFN-I)-stimulated genes
IRE1α: inositol-requiring enzyme 1
CHOP: C/EBP-homologous protein
NLRP3: STING-NOD-like receptor protein 3
MAM: mitochondria-associated ER membranes
TRAF2: tumor necrosis factor receptor-associated factor 2
Nrf2: nuclear factor erythroid 2-related factor 2
MI/R: myocardial ischemia/reperfusion
TDP43: transactive response DNA-binding protein of 43 kDa
TNF-α: tumor necrosis factor-α
CKD: chronic kidney disease
DCM: diabetic cardiomyopathy
